# CD74 and Proteases: Impact of Location on Immune and Cellular Functions

**DOI:** 10.3390/cells14241960

**Published:** 2025-12-10

**Authors:** Iztok Dolenc

**Affiliations:** Jozef Stefan Institute, Department of Biochemistry and Molecular and Structural Biology, Jamova 39, SI-1000 Ljubljana, Slovenia; iztok.dolenc@ijs.si

**Keywords:** CD74, invariant chain, MHC class II, proteases, cysteine cathepsins, cathepsin S, SPPL2a, MIF

## Abstract

**Highlights:**

**What are the main findings?**

- CD74 exists in two functional locations: within endolysosomal compartments as a chaperone and at the plasma membrane as a signaling receptor.
- Isoform-specific motifs and protease-dependent processing determine CD74 trafficking, stability, and its switch between intracellular and surface functions.

**What are the implication of the main findings?**

- The dual localization of CD74 explains how proteolytic regulation modulates both antigen presentation and receptor-mediated signaling.
- Understanding location-specific protease interactions summarizes emerging therapeutic opportunities in immune, inflammatory, and malignant diseases.

**Abstract:**

Proteases represent a diverse family of enzymes that catalyze the hydrolysis of peptide bonds, modulating numerous biological processes. Among their substrates, CD74—also known as the invariant chain—has received increased research attention due to its multifunctional roles in both innate and adaptive immunity. This review provides an overview of current knowledge on protease-mediated interactions with CD74. The protein was originally identified as a chaperone for major histocompatibility complex class II (MHC-II) molecules. Proteolytic cleavage of CD74, most notably by cathepsin S, is essential for the release of MHC-II and the initiation of antigen presentation. However, CD74 has since emerged as a central regulator of processes extending well beyond antigen presentation. More recent findings reveal that CD74, acting as a receptor of macrophage migration inhibitory factor, also participates in signaling pathways in non-immune cells, independent of its classical chaperone function. Proteolytic processing of CD74 can trigger signaling cascades that modulate gene expression, underscoring its multifunctionality. Dysregulation of CD74 cleavage and its interaction with proteases has been linked to diverse pathological conditions, including cancer and autoimmune diseases, where aberrant protease activity disrupts CD74 function and promotes disease progression.

## 1. Introduction

CD74 and proteases have garnered significant attention in scientific research due to their diverse roles in cellular processes, disease pathogenesis, and therapeutic interventions. CD74, also known as the invariant chain (Ii), is a multifunctional protein with important roles in the immune system. Initially characterized as a chaperone for major histocompatibility complex class II (MHC-II) molecules, CD74 ensures proper folding, trafficking, and stabilization of MHC-II complexes [1]. Beyond this canonical role, CD74 has emerged as a multifunctional receptor and signaling mediator, implicated in processes such as antigen presentation, lymphocyte survival, and inflammatory pathway regulation [2]. Proteases constitute a broad class of enzymes responsible for proteolytic cleavage of proteins, thereby controlling protein maturation, turnover, and activation. In CD74 biology, proteolytic processing represents a critical regulatory mechanism [3]. Sequential cleavage of CD74 governs not only antigen presentation but also soluble fragment generation and receptor-mediated signaling [4,5]. Consequently, the interplay between CD74 and proteases is increasingly recognized as a determinant of immune homeostasis [6]. However, due to the limited scientific evidence, clarifying the relationship between CD74 and proteases is therefore essential to understanding both normal immune regulation and pathological conditions [7]. Disruptions in CD74–protease interactions are linked to pathological conditions, including autoimmunity, chronic inflammation, and tumorigenesis [6]. Dysregulated proteolysis may alter antigen presentation, disrupt signaling cascades, or promote aberrant accumulation of CD74 fragments. This review aimed to synthesize current knowledge on the molecular relationship between CD74 and proteases. Furthermore, the dual role of CD74 and its processing enzymes is described in this review. Emphasis will be placed on mechanistic insights, regulatory networks, and emerging evidence that positions this axis as a potential therapeutic target in immune-mediated and malignant disorders.

## 2. Localization and Function

CD74 is broadly expressed across immune and non-immune tissues, consistent with its multifunctional role in cellular organization and immune regulation [1,2]. Its localization within distinct intracellular compartments or at membrane interfaces determines whether it functions as an endolysosomal chaperone guiding major histocompatibility complex class II (MHC class II) assembly [8] or as a membrane-associated receptor mediating signaling and cellular communication [4,6]. The following subsections describe how compartment-specific distribution and proteolytic processing govern CD74 stability, trafficking, and biological activity.

### 2.1. Endolysosomal CD74

A well-characterized role of CD74 is its function in facilitating MHC-II antigen presentation, a central process in adaptive immunity [9]. In this transmembrane complex of alpha, beta, and gamma chains, the gamma chain (CD74) acts as a chaperone protein that ensures correct folding and assembly of the MHC-II alpha and beta chains in the endoplasmic reticulum [10,11]. By binding to these chains, CD74 stabilizes the nascent MHC-II heterodimers and prevents premature peptide binding. Additionally, CD74 directs the complex into the endosomal/lysosomal pathway, where CD74 degrades under the control of acidic pH (further elaborated upon in chapter 6.1), leaving a small fragment, known as class II-associated invariant chain peptide (CLIP), which occupies the peptide-binding groove [12]. This placeholder peptide is later exchanged for antigenic peptides through the action of human leukocyte antigen (HLA)-DM, enabling proper presentation of foreign antigens on the cell surface. Hence, CD74 serves both as a molecular scaffold and a regulator, ensuring immune specificity and preventing inappropriate self-peptide loading.

### 2.2. Cell Surface CD74

Our understanding of CD74 has evolved beyond its classical roles in the immune system, with emerging evidence revealing its involvement in diverse physiological and pathological processes, particularly various inflammatory processes. CD74 acts as a regulator of hematopoietic stem cell maintenance [13], influencing B cell survival, proliferation, and differentiation through interactions with cytokines and growth factors [14,15]. As a signaling receptor for the proinflammatory cytokine macrophage migration inhibitory factor (MIF), CD74 triggers intracellular signaling pathways involved in immune responses, inflammation, neuroinflammation, atherosclerosis, cell migration, and wound healing [4,16,17,18], as well as tissue repair in various parts of the body [19,20]; it is also involved in skin-aging mechanisms [21]. Dysregulation of CD74 expression and function has been associated with several autoimmune diseases including rheumatoid arthritis [22], systemic lupus erythematosus [23], inflammatory bowel disease [14,19], and autoimmune thyroid diseases [24], highlighting its importance in the immune system. CD74 expression levels are significantly upregulated in most cancers than in normal tissues [25]; CD74 supports the accumulation and function of regulatory T cells in tumors [26]. CD74 is also expressed in microglia and astrocytes, where it participates in neuroinflammatory and neurodegenerative processes [27,28]. A soluble variant of CD74 was discovered in autoimmune liver disease, in which membrane-truncated CD74 is released after proteolytic processing [29]. Extracellular cathepsins [30] may interact with CD74 at the cell surface; however, such interactions occur predominantly in neutral pH conditions. Contrastingly, within acidic endosomal or lysosomal compartments, the enzymatic properties of cathepsins can differ considerably, which may alter both the likelihood and the outcome of their interactions with CD74.

## 3. Gene Structure and Expression Control Mechanisms

**Figure 1:**
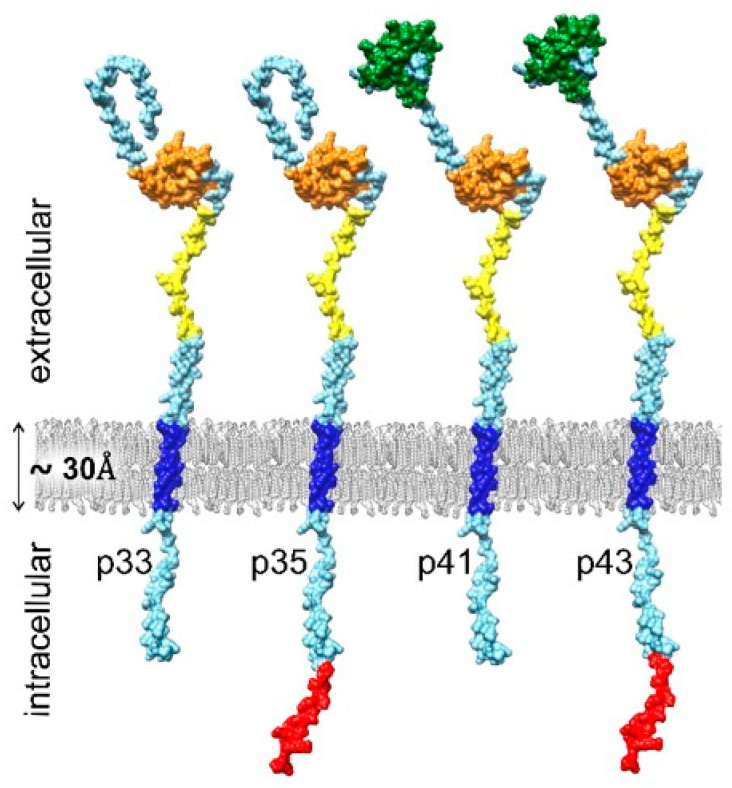
The domain architecture of all four splice variants of CD74. 3D scheme is based on models of CD74 single chains prepared using an algorithm for protein structure prediction, trRosetta [36]. The splice differences are indicated in red (N-terminal region) and green (thyroglobulin type 1 domain inhibitor). The transmembrane region, which measures approximately 30 Å [37], is colored blue, while the CLIP region is highlighted in yellow and the trimerization region is denoted in orange. CLIP, class II-associated invariant chain peptide; trRosetta, transform-restrained Rosetta.

Understanding the gene structure of CD74 is of utmost importance to elucidate its functional diversity and regulatory mechanisms. The CD74 sequence was determined from a gene expressed in chronic lymphocytic leukemia. A cDNA consisting of a sequence of 1282 nucleotides, along with approximately 160-nucleotide-long poly A tail, was identified [31]. The gene responsible for encoding human CD74 is located within a specific region of chromosome 5 in humans. This gene spans approximately 11 kb and is composed of 8–9 exons. Each exon contributes distinct functional domains to the CD74 protein [31,32,33] (Figure 1). Exon 1 encodes an N-terminal intracellular cytoplasmic tail [34]. In longer isoforms (p35/p43), an upstream AUG adds 16 amino acids. A part of exon 2 contains the coding of a single transmembrane segment peptide region within the membrane of the endosomal and lysosomal compartments, or alternatively, a cell surface membrane. Exons 2–4 contain the coding for the CLIP peptide, which overlaps with the protein part responsible for the interaction with MIF [4]. The trimerization of CD74 is essential in the region coded by exons 4–6 [35]. Furthermore, exon 6b, located between exons 6 and 7, encodes a thyroglobulin type-1 domain that is incorporated into the p41 and p43 isoforms through alternative splicing [32,34].

Oncogenic fusion proteins resulting from chromosomal rearrangements or translocations are distinctive features of cancer development. Whole-genome sequencing revealed recurrent CD74 genomic rearrangements, implicating it in the pathogenesis of rare lymphomas [38]. Fusion proteins play crucial roles in driving abnormal signaling pathways and promoting cellular proliferation. Among the various fusion proteins associated with cancer, those involving CD74 have attracted considerable attention because of their involvement in tumor formation and potential therapeutic implications. In their study, Vargas and Pantouris highlighted several intriguing oncogenic fusion proteins, namely CD74-PDGFRB, CD74-ROS1, CD74-NTRK1, CD74-NRG1, and CD74-NRG2α, which exhibit 16 variants [33]. Triple oncogenic fusions involving CD74 (PDGFRB/ROS1/NTRK1) were identified in lung tumors [39]. An assay for the detection of gene fusion CD74-ROS1 has been developed [40].

Transcriptional regulation of CD74 expression is mediated by diverse transcription factors and signaling cascades responsive to extracellular stimuli, including cytokines, growth factors, and microbial products [41]. Cytokines such as transforming growth factor β [42] and interferon-γ [43,44] enhance CD74 expression, while MHC II transactivator (CIITA) serves as a critical activator. During viral infections, CIITA-induced expression of the p41 isoform prevents cathepsin-mediated glycoprotein processing, thereby blocking Ebola virus and severe acute respiratory syndrome (SARS)-like coronavirus entry [45]. In the context of immune regulation, altered CD74 expression has been observed in regulatory T cells within tumors [26] and during viral infections [46]. Notably, CD74 isoforms p33 and p35, but not p41 or p43, can inhibit *Enterovirus* D68 replication [47], suggesting isoform-specific antiviral activity.

**Figure 2:**
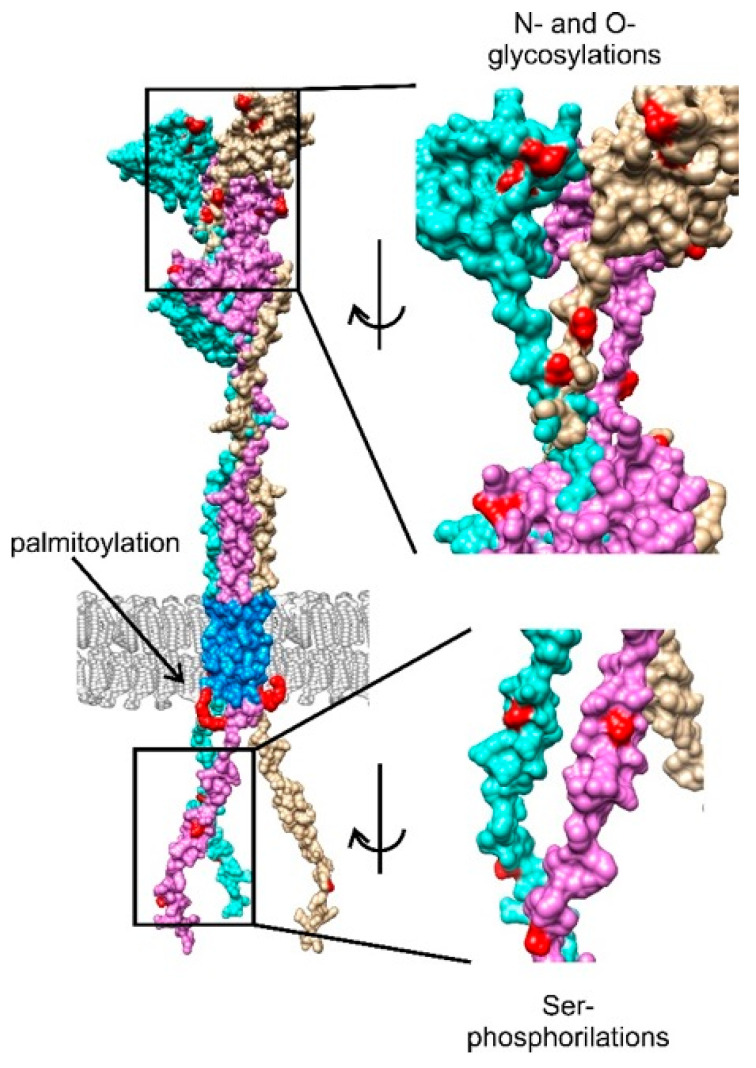
3D scheme of the CD74 trimer (p43 splice variant). The positions of post-translational modifications (N- and O-glycosylation, palmitoylation, and Ser phosphorylation) are marked in red. Sections of CD74 related to glycosylation and phosphorylation modifications are enlarged and rotated to enhance visibility and clarity. Each chain is uniquely colored. The transmembrane region is stained blue. The 3D scheme is based on the model of the CD74 single chain, using an algorithm for protein structure prediction, trRosetta [36]. CD74 trimer assembly was prepared using the M-ZDOCK tool, which facilitates symmetric multimer docking [54]. trRosetta, transform-restrained Rosetta.

Post-translational modifications critically regulate CD74 function, localization, and interactions [48]. Glycosylation is the most extensively characterized modification (Figure 2). Both N-linked and O-linked glycosylation were demonstrated early [49], with functional roles in protein stability, trafficking, and antigen presentation [50]. N-linked glycosylation protects against premature proteolysis in neutral environments, while structural studies revealed two N-glycosylation sites (Asn242 and Asn256) in the thyroglobulin type-1 domain [51]. According to UniProt (P04233), additional N-linked sites occur at Asn130 and Asn136, with O-linked glycosylation at Thr203 and Ser281. Arneson et al. (2007) further identified an O-linked chondroitin sulfate attachment at Ser282, promoting rapid transport from the trans-Golgi to the cell surface [52]. Sialylation of N- and O-glycan chains indicates processing through the trans-Golgi apparatus [53].

Phosphorylation also contributes to CD74 regulation (Figure 2). Serine residues in the N-terminal domain undergo phosphorylation, facilitating endoplasmic reticulum exit and subsequent endosomal transport of CD74 within the MHC-II complex [55,56]. Although CD74 lacks tyrosine and threonine residues in its intracellular tail, downstream signaling is mediated via CD44 and MIF interactions, leading to Src kinase family activation and phosphorylation of ERK1/2 [57,58].

Additionally, CD74 undergoes palmitoylation at Cys27 (Figure 2), adjacent to the intracellular membrane surface, enhancing membrane association [59]. While palmitoylation modulates protein–membrane interactions, transmembrane proteolysis of CD74 can occur independently of this modification [60]. Together, these post-translational modifications integrate structural stability with immune regulation, signaling, and viral restriction.

## 4. Protein Domains of CD74 and Their Multifunctional Roles

Primary segmentation of various areas shows three distinct domains with different functions: an extracellular domain, transmembrane domain, and intracellular domain (ICD) [2] (Figure 1). These diverse functions are attributed to their unique structural and functional domains. The identification of these structural and functional elements has enabled researchers to associate motifs with important functions in diverse biological processes such as antigen presentation, immune regulation, and cell signaling.

### 4.1. Extracellular Domain

The largest part of the CD74 protein is its extracellular domain [61]. This region contains several functional motifs and binding interfaces. This domain harbors the elements that enable CD74 to assemble into homotrimers [62,63,64]. CD74 plays a central role in antigen presentation by interacting primarily with MHC-II molecules through its transmembrane and extracellular domains [37]. Trimers can associate with three MHC-II heterodimers (αβ), creating a complex structure of a nine-subunit complex [65,66]. A critical feature of this interaction is the CLIP region (within the p41 isoform amino acids 97–120), which transiently occupies the peptide-binding groove of MHC-II until displaced by antigenic peptides [67,68,69]. Beyond its chaperone function, CD74 also engages with a variety of signaling molecules and adaptor proteins, thereby modulating intracellular signaling cascades. Notably, recent findings in IRF8-mutant B-cell lymphoma demonstrate that CD74 loss disrupts MHC-II antigen processing and presentation, leading to impaired CD4^+^ T-cell activation and facilitating immune evasion [70]. Another region of major functional importance in CD74 is the thyroglobulin type-1 domain (thyropin), which is present in p41 and p43 isoforms. This domain functions as an endogenous inhibitor of selected cathepsins [32,71].

The surface expression of CD74 does not rely on the simultaneous expression of MHC-II [53]. Gastric epithelial cells show a high level of surface protein expression, which is polarized to the apical surface [72]. When modified with chondroitin sulfate, CD74 promotes the rapid transport of proteins from the trans-Golgi network to the cell surface [52], generating distinct surface pools of the protein as a receptor. At the cell surface, CD74 binds MIF [4,73] and its structural homolog D-dopachrome tautomerase (MIF-2, or D-DT) with high affinity (binding interaction *K_D_* of 1.40 nM and 5.4 nM, respectively) [74]. Binding of MIF to the extracellular domain of CD74 activates downstream ERK1/2 signaling [4]. CD74 signaling in connection with MIF has been comprehensively reviewed [19]. Notably, small-molecule inhibitors targeting MIF effectively block its interaction with CD74, offering a promising strategy for therapeutic potential [75]. Notably, the stimulation of the B-cell lymphoblastoid line with anti-CD74 antibody induces a signaling cascade similar to MIF response, demonstrating that surface CD74 functions as a survival receptor [76]. Through MIF interaction, CD74 activates intracellular pathways including ERK1/2, AKT, and NF-κB, which contribute to immune responses and inflammatory processes [4,6,19,77,78]. Small molecule inhibitors of MIF can block the interactions between MIF and CD74 [75]. Moreover, CD74 can form complexes with other CXC chemokine receptors such as CXCR2, CXCR4, and CXCR7, thereby amplifying its involvement in inflammatory responses and cell migration [19].

Additionally, the cytoplasmic tail of CD74 functions as a critical platform for the recruitment of adaptor proteins and cytoskeletal elements, thereby facilitating receptor internalization and the initiation of downstream signaling pathways. Similar to interaction with MIF, CD74 has also been shown to associate with tissue inhibitor of metalloproteinases-1 (TIMP1) in a mechanistically analogous manner [79,80]. These two molecules interact with each other within the cellular environment of breast cancer cells [81]. The interacting part of the inhibitor is the N-terminal domain. The domain harbors antiproteolytic activities and triggers intracellular zeta chain–associated protein kinase-70 (ZAP-70) phosphorylation [82]. Notably, the expression of CD74 is significantly associated with the expression of ZAP-70 in patients diagnosed with B-cell chronic lymphocytic leukemia [83], which could be explained by the CD74–TIMP1 interaction.

### 4.2. Transmembrane Domain

The transmembrane region of CD74 is a 24 amino acid-long single-pass α-helix and drives trimerization, a critical step for stabilizing MHC-II αβ heterodimers and facilitating their export from the endoplasmic reticulum to endosomal compartments [10,84,85]. The transmembrane region domain also influences lateral segregation into lipid microdomains where CD74 interacts with co-receptors such as CD44 [58,84,86]. Moreover, structural integrity of the transmembrane region is required for proper clustering and surface stability of CD74, which underpins its roles both as a MHC-II chaperone and as a receptor for external ligands. Mutations or disruptions in the transmembrane region hydrophobicity compromise oligomerization and membrane localization, reducing antigen presentation efficiency and impairing immune regulation [61].

### 4.3. Intracellular (Cytoplasmic) Domain

The cytoplasmic tail, located at the N-terminal end of CD74, is essential for intracellular signaling and protein trafficking [87,88]. Isoforms p33 and p41 share a short 30–amino acid cytosolic N-terminus, whereas p35 and p43 contain an extended N-terminal domain (16 additional amino acids). This part incorporates an arginine-based atypical sorting signal for endosomal compartments coupled to cell surface transport [89,90].

Within this domain, there are sites for potential phosphorylation and protein interaction motifs that control the signaling pathways and trafficking processes mediated by CD74. The downstream signaling pathways initiated by CD74–MIF interaction are conditioned by the binding of CD74 to CD44, a cell adhesion molecule that plays a crucial role in cell proliferation, migration, and metastasis [91,92,93]. CD74 alone is sufficient to mediate extracellular MIF binding to cells. However, in the absence of CD44, or even when only its N-terminal domain is missing, MIF is unable to trigger phosphorylation of ERK1/2 kinases. The authors indicate that MIF induces the serine phosphorylation of the CD74 intracytoplasmic domain in a CD44-dependent manner [58].

Beyond serving as a signaling hub, the cytoplasmic domain of CD74 harbors specific motifs that govern its intracellular routing, ensuring the proper balance between ligand-induced signaling and endocytic transport. Particularly, it contains leucine-based sorting motifs that are specifically recognized by clathrin adaptor complexes AP-1 and AP-2 [94]. Clathrin adaptor complexes mediate cargo selection and vesicle formation in intracellular trafficking. Through mutational analysis, they showed that these motifs are essential for directing CD74 trafficking into the endocytic pathway, thereby regulating MHC-II transport.

### 4.4. Mechanistic Link Between Intracellular Trafficking and Surface Receptor Function of CD74

The dual behavior of CD74—as an intracellular chaperone and a plasma membrane receptor—appears to result from compartment-specific processing and sequence-dependent sorting mechanisms. Newly synthesized CD74 molecules trimerize in the endoplasmic reticulum and associate with nascent MHC-II molecules to prevent premature peptide loading [84,85]. Export from the endoplasmic reticulum depends on serine phosphorylation and arginine-based motifs in the cytosolic tail that facilitate trafficking to the Golgi apparatus [86]. Following endoplasmic reticulum exit, dileucine-based motifs engage adaptor proteins AP-1 and AP-2, directing the protein into endosomal and lysosomal compartments [87,88]. Within these acidic vesicles, cysteine cathepsins—mainly cathepsins S and L—sequentially degrade the luminal portion of CD74 to generate CLIP [8]. The remaining membrane-anchored fragment is then cleaved by the intramembrane protease SPPL2a, completing an intracellular domain (ICD) that can translocate to the nucleus and contribute to transcriptional regulation [95,96].

In addition to the intracellular degradative route, a distinct trafficking pathway delivers a subset of CD74 molecules to the plasma membrane [19]. This surface-directed pool bypasses lysosomal degradation and remains signaling-competent, enabling interaction with MIF and related ligands [73].

Distinct sorting motifs and post-translational modifications acquired during transport, together with isoform-dependent variations in the N-terminal sequence, define this alternative trafficking route [2]. Isoforms p33 and p41 possess a short 30-amino-acid cytosolic tail, whereas p35 and p43 contain an additional 16-amino-acid N-terminal extension enriched in arginine and serine residues. Phosphorylation within this segment promotes endoplasmic reticulum exit and favors trafficking toward the plasma membrane, resulting in increased cell-surface localization [86,89,90]. Once at the cell surface, sequence-dependent variation in CD74 modulates protease accessibility—particularly to extracellular cathepsins—thereby regulating receptor stability, turnover, and signaling duration. SPPL2a-mediated cleavage also contributes to the turnover of surface CD74, regulating receptor availability and signal propagation [97].

Although this model is supported by biochemical and sequence-based evidence, experimental validation of how N-terminal variation governs localization and surface stability across all four CD74 variants remains limited. The subcellular distribution between endolysosomal and plasma membrane compartments ultimately determines whether CD74 functions as a chaperone or as a signaling receptor involved in immune regulation. The structural organization, interacting partners, post-translational modifications, and related functional implications of CD74 are summarized in Table 1.

## 5. Proteolytic Interaction and Functional Implications

**Figure 3:**
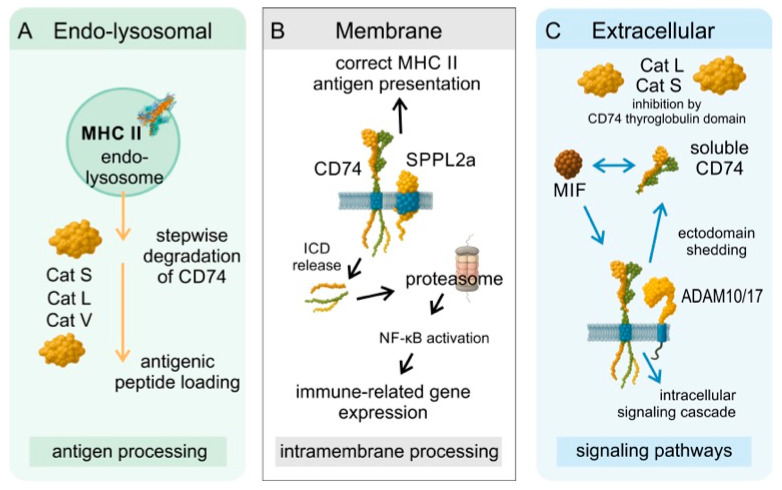
Proteolytic regulation of CD74 across cellular compartments. Schematic overview of CD74 processing by proteases in distinct environments. (**A**) In the endolysosomal compartment, cathepsins S, L, and V mediate stepwise degradation of CD74 and release of the CLIP fragment for MHC-II loading. (**B**) The intramembrane protease SPPL2a cleaves membrane-bound CD74, releasing the intracellular domain (ICD) involved in signaling. (**C**) At the cell surface and in the extracellular space, ADAM10/17 and secreted cathepsins induce ectodomain shedding and generate soluble CD74, which modulates MIF-mediated signaling and cathepsin activity. Arrows indicate proteolytic direction and outcome (degradation, activation, or inhibition).

The interaction between CD74 and proteases represents a multifaceted and complex regulatory network shaped by two biological functions of CD74—as an intracellular chaperone involved in antigen presentation and as a cell-surface receptor that triggers signaling pathways. Proteases influence these roles in distinct ways, depending on their type and site of action. Intramembrane protease signal peptide peptidase-like 2a (SPPL2a) acts on membrane-anchored remnants of CD74. Endosomal/lysosomal proteases, such as cathepsins, initiate stepwise cleavage of CD74 in an acidic environment, controlling its stability and turnover during antigen presentation. Extracellular and secreted proteases, including matrix metalloproteinases (MMPs) and cathepsins, can modulate CD74 at the plasma membrane at neutral pH. This division highlights how distinct groups of proteases engage with CD74 in context-dependent manners, orchestrating both structural processing, functional regulation, and coordinating cellular responses. The principal proteolytic events and their compartmental localization are schematically summarized in Figure 3.

### 5.1. Intramembrane Cleavage of CD74

SPPL2a is a member of the GxGD intramembrane protease family [97]. It is primarily localized to late endosomes and lysosomes, compartments where CD74 processing occurs [2,100,101,102]. It controls the degradation of the membrane-bound CD74 [111]. The enzyme’s N-terminal PA domain acts as a targeting signal required for endosomal localization [112]. Upon contact with CD74, the transmembrane regions of SPPL2a surround the substrate and position it in the catalytic site, where cleavage occurs and the CD74 ICD is released [100]. The main cleavage site is situated between residues Y52 and F53 within the CD74 transmembrane segment [60]. Inhibition or deficiency of SPPL2a prevents proper CD74 processing, resulting in the accumulation of full-length protein and impaired MHC-II antigen presentation [95]. Following release, the ICD is degraded by the proteasome [96], consistent with cytosolic proteases rapidly targeting peptides longer than 14 amino acids [113]. However, the CD74 ICD can interact transiently with transcription factors such as RUNX or NF-κB, translocating to the nucleus to regulate immune-related gene expression [88]. Loss-of-function mutations in SPPL2a, identified in patients with Mycobacterium bovis disease, cause pathological accumulation of CD74 fragments in lymphoid cells [111]. Moreover, the CD74 ICD is a heme-binding protein, and SPPL2a-mediated cleavage modifies its heme ligation properties, shifting from stable to low-affinity binding [114].

### 5.2. Endosomal Degradation of CD74

Cysteine cathepsins are lysosomal proteases essential for protein turnover, antigen presentation, and tissue remodeling. They belong to the papain-like family, defined by a catalytic cysteine residue. Eleven members exist in humans: cathepsins B, C, H, F, K, L, O, S, V, W, and X [115]. While most function as monomers, structural diversity occurs: cathepsin C forms tetramers [116], cathepsin X exists as a dimer [117], and cathepsin K dimerizes in the presence of oligosaccharides [118]. These proteases act within lysosomes, endosomes, and extracellular compartments, and dysregulated activity contributes to cancer, autoimmune disease, cardiovascular pathology, and neurodegeneration.

During the transport of MHC-II to endosomal compartments, CD74 (invariant chain) functions as a chaperone, stabilizing MHC-II and shielding its peptide-binding groove [1]. The transport of CD74 to the plasma membrane is not influenced by the presence of MHC-II molecules in the endocytic pathway, as this proportion of CD74 is independently transported to the plasma membrane [53]. However, for efficient peptide binding, MHC-II molecules must reach antigen-processing compartments and CD74 must be degraded within endosomes [1]. This process involves the degradation of CD74, primarily mediated by cathepsins, with cathepsin S acting as the principal protease. It is enriched in antigen-presenting cells, optimally active at acidic pH, and essential for invariant chain removal [8,119]. Together with cathepsin L, it cleaves CD74 [103,120], and deficiency in either enzyme severely disrupts humoral immunity and T cell selection [121]. Cleavage sites remain undefined, though in vitro studies with recombinant CD74 revealed distinct cathepsin S-generated fragments [122]. Cathepsin V can substitute for cathepsin L [104], while cathepsin F, cathepsin B, and legumain are largely dispensable [123]. This redundancy ensures robustness, but cathepsin S holds a unique nonredundant role.

Upon endosomal acidification, γ-interferon-inducible lysosomal thiol reductase (GILT) activates cathepsin S [124]. Active cathepsin S cleaves CD74 within the region spanning the CLIP and the trimerization domain, resulting in minimal impact on the oligomerization of the HLA-DO/CD74 complex [37]. This proteolysis liberates CLIP from the MHC-II binding groove, thereby exposing the peptide-binding site. However, efficient peptide loading does not occur spontaneously. The nonclassical MHC-II molecule HLA-DM facilitates the release of residual CLIP and catalyzes the exchange for high-affinity antigenic peptides derived from exogenous proteins. This peptide exchange ensures the stability of the peptide–MHC-II complex and promotes optimal presentation to CD4^+^ T cells. HLA-DO, expressed in B cells and thymic epithelial cells, can further modulate this process by regulating HLA-DM activity and thus fine-tuning peptide selection [125]. Although cysteine cathepsins dominate CD74 degradation, other proteases may contribute. Inhibition of aspartic cathepsins D and E with pepstatin A blocks CD74 removal and MHC-II maturation [126]. However, as pepstatin A does not readily cross membranes without lipid modification, intramembrane proteases such as SPPL2a are unlikely to be affected [127]. These results suggest a supporting role for aspartic proteases, possibly through cross-regulation of cysteine cathepsins.

### 5.3. Cell Surface CD74 Can Interact with Extracellular Proteases

CD74 localization is not only in acid vesicles. It can be transferred to the plasma membrane in the absence of other MHC-II molecules, as indicated by the research on lymphoblastic cell lines CEM.T1 and CEM.T2 [53]. This clearly suggested localization of free CD74 molecules on the cell surface. The importance of CD74 molecules on the cell surface has been demonstrated through interactions with extracellular proteins at neutral pH, triggering cellular responses.

The mechanism of CD74 proteolysis in the extracellular space remains largely unknown. Studies have frequently reported the ability of cathepsins to cleave CD74 on the cell surface, but these claims are primarily derived from observations of cleavage events occurring in slightly acidic endosomes during antigen presentation [8]. Cysteine cathepsins are secreted from cells and can act extracellularly, suggesting a potential role in the degradation of cell surface proteins [30]. Proteolytic cleavage of cell surface CD74 generates soluble CD74 (sCD74). The extracellular domain of transmembrane CD74 can be shed into the extracellular space [128], and sCD74 has been detected in diverse pathological contexts, including cardiac disease [129], acute lung injury [130], and melanoma [5]. This ectodomain shedding is protease-dependent, with MMPs A Disintegrin And Metalloproteinase (ADAM)10, ADAM17, and cysteine proteases implicated in CD74 cleavage [5].

ADAM MMPs are a family of membrane-anchored enzymes with prominent sheddase activity that have dual functions: proteolytic activity and cell signaling [131]. They are characterized by having both a disintegrin and a MMP domain. The MMP domain is responsible for their proteolytic activity, enabling them to cleave various cell surface proteins, while the disintegrin domain is involved in interactions with integrins and other cell adhesion molecules [132]. The mechanism of ADAM10 function can be understood from the overall enzyme architecture based on the X-ray crystal structure of the complete enzyme ectodomain. The catalytic domain precedes the transmembrane region of the protein [133]. ADAM MMPs can modulate the abundance and structural integrity of CD74 at the plasma membrane, thereby influencing its receptor functionality and downstream signaling pathways [5]. Notably, the type of protease responsible for the cell-surface cleavage of CD74 depends on the cell type. Further research is required to identify the protease responsible for producing sCD74. However, researchers have shown that SPPL2a is not involved in the process of ectodomain shedding, as knockdown of the intramembrane-cleaving protease did not change the release of sCD74 [5].

Cysteine cathepsins play an important role in breaking down CD74. However, cathepsin S can be weakly inhibited by a 64-residue fragment derived from the p41 or p43 splice variants. This fragment, known as the thyroglobulin type I domain or thyropin inhibitor [71], modulates the activity of some cathepsins [32,134]. Multiple isoforms of CD74 are statistically integrated into trimeric structures, indicating that different splice variants of CD74 combine to form trimers. This structural organization increases the likelihood that a thyropin inhibitor is present within the CD74 trimer [135]. In the absence of the thyropin domain, the CD74 complex can be effectively degraded by cathepsin L [136]. The interaction between cathepsin S and inhibitory factor p41 results in reversible inhibition of the enzyme, characterized by an equilibrium inhibition constant (Ki) of 208 nM [134]. This suggests relatively weak inhibition, implying that cathepsin S is directed away from the C-terminus of the chain. Cathepsins H and L are also inhibited by thyropin domain, with Ki values 5.3 nM and 1.7 pM, respectively [109]. The strong inhibition of cathepsin L highlights its significance. The interaction of the inhibitory p41 fragment with the active cathepsin L allows extracellular accumulation of the active enzyme [106]. Notably, cathepsin L is an unstable enzyme at neutral pH, with an inactivation rate constant (kinact) of 0.15 s-1 at pH 7.4 and 37 °C [137]. However, substrates and protein inhibitors such as cystatins can stabilize the active conformation of cathepsin L [138,139]. The stabilization of cathepsin L in complex with the CD74 inhibitor domain enables the isolation of cathepsin L from the human kidney [140]. The presence of the soluble cathepsin L-CD74 inhibitor complex suggests that the inhibitory domain can be cleaved from the rest of the molecule, although the specific enzyme responsible for this cleavage remains unidentified. The inhibition of cathepsin L by CD74 raises the possibility of cathepsin L activity in the extracellular environment [30,106]. The extracellular activity of cathepsin L may have significant implications for various physiological and pathological processes. Beyond its canonical role, cathepsin S also influences transcriptional regulation. Through CD74 cleavage, which triggers CD74-dependent signaling (via NF-κB), it modulates chemokine processing and alters expression of CCL2 [141]. These findings highlight its dual role in antigen processing and immune regulation.

Overall, these findings underscore the complex regulatory mechanisms involving cathepsins and CD74, highlighting the intricate balance between enzyme activity and inhibition in maintaining cellular homeostasis and contributing to disease pathogenesis. Infectious pathogens can also exploit CD74 as an entry or signaling partner. *H. pylori* urease B binds CD74 to induce inflammation, while HIV proteins gp41 and Vpu interact with CD74 to modulate immune recognition [142,143,144]. Extracellular cathepsin L plays a crucial role in the pathogenesis of lethal sepsis [145]. Elevated levels of cathepsin L have been observed in the blood plasma of patients, both before and after surgical resection, indicating its significant involvement in the body’s response to surgical stress [146]. There are other correlations with CD74. Cathepsin L contributes to a proinflammatory profile and exacerbates inflammatory responses [147]. In obesity-related diseases, cathepsin L secretion is enhanced, which is closely associated with increased inflammation, suggesting a link between metabolic disorders and heightened inflammatory states [147].

Cysteine cathepsins, including cathepsin L, have been implicated in the development and progression of cardiovascular diseases. This is particularly evident in older adults, where the incidence and severity of these conditions are more pronounced [148,149]. The role of CD74 in cardiovascular disease is significant, with CD74 regulating extracellular cathepsin L activity. This regulatory mechanism suggests that cathepsin L plays an active role in the pathology of cardiovascular conditions because of the influence of CD74 [7,106]. Notably, sepsis, postoperative inflammatory responses, obesity-related inflammation, and cardiovascular diseases are high-risk factors for severe COVID-19 infection. The association between cathepsin L, CD74, and COVID-19 is particularly notable. The p41 isoform of CD74 inhibits viral entry by blocking cathepsin L-mediated processing of viral proteins, such as the Ebola glycoprotein, which also extends to coronaviruses, including SARS-coronavirus 2 [150,151,152]. This multifaceted involvement underscores the critical role of cathepsin L and CD74 in various diseases and highlights their potential as therapeutic targets in managing severe COVID-19 infections [45]. Further research is required to elucidate the specific mechanisms by which cysteine cathepsins contribute to CD74 degradation on cell surfaces.

## 6. Potential Therapeutic Targets in Proteolysis of CD74

Targeting the proteolytic processing of CD74 has emerged as a rational therapeutic strategy across cancer, autoimmune, and inflammatory diseases. CD74 functions both as an accessory molecule for MHC-II and as a signaling receptor for MIF. Dysregulated CD74 expression or turnover is linked to pathological immune activation, tumor progression, and resistance to therapy [153,154,155]. By identifying the specific enzymes or pathways involved in the degradation of CD74, researchers can intervene and modulate these processes to regulate CD74 levels or activity.

Mechanistically, CD74 undergoes stepwise proteolysis. Its luminal domain is trimmed by cysteine cathepsins, notably cathepsin S, and subsequently, the intramembrane protease SPPL2a releases the cytoplasmic fragment that influences downstream signaling [156,157,158,159]. Modulating these cleavage steps provides opportunities for intervention: inhibition of cathepsin S in malignant B cells alters antigen processing and MHC-II maturation, triggering anti-tumor immune responses and reducing tumor growth [156]. Similarly, pharmacological SPPL2a blockade can reshape antigen-presenting cell function, although genetic evidence shows that SPPL2a loss in humans leads to dendritic cell and B-cell defects with increased infection risk, highlighting the need for caution [160].

Cysteine cathepsins themselves are druggable targets [161]. Despite the abundance of chemical inhibitors, only a few have reached clinical testing, with mixed outcomes [162,163,164]. Recent approaches seek to overcome these limitations, such as engineering non-natural peptide inhibitors (NNPIs) with reactive warheads and coupling them to antibodies for cell-specific drug delivery [165]. This strategy may minimize off-target toxicity while enhancing therapeutic precision.

Beyond protease inhibition, direct targeting of CD74 has also been pursued. The anti-CD74 monoclonal antibody milatuzumab has shown preclinical activity in B-cell malignancies and early clinical exploration in hematologic cancers and autoimmune disease [166]. In solid tumors, CD74 expression correlates with inflamed tumor microenvironments and favorable responses to immune checkpoint inhibitors, positioning CD74 both as a therapeutic target and as a biomarker [167].

Overall, CD74-centered interventions can be pursued at several mechanistic levels. At the plasma membrane, therapies aim to block MIF–CD74 interactions or reduce receptor abundance through monoclonal antibodies, thereby limiting downstream signaling. Within the endolysosomal pathway, inhibition of proteolytic processing by cathepsin S or SPPL2a offers a strategy to reprogram antigen presentation and reshape immune responses. Additionally, combination approaches that integrate antibody-based targeting with selective protease inhibition, or that simultaneously modulate multiple pathways, hold promise for achieving greater efficacy and therapeutic precision while minimizing systemic toxicity. An integrative overview of CD74 regulatory mechanisms, signaling pathways, disease associations, and therapeutic targeting approaches is presented in Table 2.

## 7. Conclusions

This review underscores the central role of CD74 and proteases in regulating immune responses and cell signaling, highlighting their intricate and reciprocal interplay. Proteases cleave CD74 in a stepwise manner, releasing defined fragments that directly influence antigen presentation and downstream signaling pathways. Importantly, CD74 itself exists in two functional contexts: at the cell surface under neutral pH, where it serves as a receptor for MIF and other ligands, and within the endosomal–lysosomal compartment at acidic pH, where it functions as an invariant chain regulating MHC-II loading and undergoes proteolytic processing. This dual localization underscores the complexity of CD74 biology and explains how proteases exert context-dependent effects on its activity. Clarifying these mechanisms not only refines the understanding of immune system dynamics but also lays the groundwork for innovative therapeutic approaches in cancer, autoimmunity, and inflammatory diseases.

## Figures and Tables

**Table 1:** Structural organization, interacting proteins, post-translational modifications, and functional relevance of CD74.

CD74 Region/ Domain	Key Functions	Known Interacting Proteins/Proteases	Post-Translational Modifications	Functional Significance/Related Diseases
Cytoplasmic tail	Endosomal sorting; signaling adaptor; internalization motifs	CD44 [58]	Serine phosphorylation [58,98]	Regulates receptor signaling; involved in immune-cell activation and chronic inflammation [55,99]
Transmembrane domain	Anchors CD74 in membrane; site of intramembrane proteolysis	SPPL2a [100,101,102]	Cleavage by SPPL2a → ICD release [100]	Generates ICD influencing NF-κB/RUNX activity [88]
Endolysosomal (intraluminal) domain	Located inside endosomal and lysosomal compartments; chaperone for MHC-II assembly and trafficking; contains CLIP sequence and Tg domain that inhibits cathepsins	MHC-II α/β chains, HLA-DM, cathepsins L and S [8]	Sequential degradation by cathepsins S, L, V [51,103,104]; N-/O-glycosylation [2]; chondroitin-sulfate attachment [52]	Controls antigen presentation and protease inhibition; essential for MHC-II maturation; altered cleavage linked to immune dysfunction and tumorigenesis [99]
Cell-surface domain	Receptor for MIF, MIF-2, and TIMP-1; substrate for ectodomain shedding and proteolytic processing	ADAM10/17 [5]; cathepsins S [105] and L [106] TIMP-1 [81]	N-/O-glycosylation [2]; palmitoylation [107]; phosphorylation [55]	Activates ERK1/2, AKT, and NF-κB pathways [6]; regulates cell proliferation and inflammatory responses [6]; overexpressed in cancer and autoimmune diseases [108]
Soluble CD74	Circulating fragment acting as modulator of MIF signaling	MIF [58]; cathepsin L [109]	Proteolytic shedding by ADAM10/17 [5]	Functions as decoy receptor [110]; may attenuate inflammatory or tumor-promoting effects [5]

**Table 2:** Integrative overview of CD74 regulatory network, associated signaling pathways, disease links, and therapeutic targeting strategies. Summary of the regulatory mechanisms, signaling pathways, disease associations, and therapeutic targeting approaches involving CD74.

Aspect	Description
Upstream Regulation of CD74 expression	Cytokines: IFN-γ, TNF-α, IL-6 → transcriptional upregulation of CD74 [42,43,44,168]. Proteases: Cathepsins S, L, V; ADAM10/17; SPPL2a → post-translational processing [5,30,100]. Inflammatory and stress stimuli enhance CD74 expression [18,25,169].
Functional Hub	Acts as invariant chain (MHC class II chaperone) [8]. Functions as cell-surface receptor for MIF, D-DT, and TIMP1 [4,74,79]. Integrates immune, stress, and proteolytic signaling pathways [6,7,19].
Signaling Pathways and Disease Associations	Signaling: NF-κB, ERK1/2, PI3K/AKT → cell survival and inflammation [6,14,170]. Immune regulation: antigen presentation, macrophage activation [8,9,171]. Cancer: proliferation, metastasis, immune evasion [25,172,173,174]. Autoimmune/Inflammatory diseases [6,14,153]. Neurodegeneration, Alzheimer’s disease [175].
Therapeutic Targeting of CD74 Networks	Anti-CD74 antibodies: milatuzumab, STRO-001 [166,176]. MIF inhibitors: ISO-1, ibudilast, neutralizing antibodies [177,178,179]. Cathepsin S inibitors [105,164]. SPPL2a inhibitors [158]. ADAM 10/17 regulation [180,181]

## Data Availability

No new data were created or analyzed in this study. Data sharing does not apply to this article.

## Abbreviations

The following abbreviations are used in this manuscript:

MHC-II	major histocompatibility complex class II
Ii	invariant chain
MIF	macrophage migration inhibitory factor
SPPL2a	signal peptide peptidase–like 2a
CLIP	class II-associated invariant chain peptide
CIITA	class II major histocompatibility complex transactivator
HLA	human leukocyte antigen
